# Effectiveness of score card-based antenatal risk selection, care pathways, and multidisciplinary consultation in the Healthy Pregnancy 4 All study (HP4ALL): study protocol for a cluster randomized controlled trial

**DOI:** 10.1186/1745-6215-16-8

**Published:** 2015-01-06

**Authors:** Amber A Vos, Sabine F van Voorst, Adja JM Waelput, Lieke C de Jong-Potjer, Gouke J Bonsel, Eric AP Steegers, Semiha Denktaş

**Affiliations:** Division of Obstetrics and Prenatal Medicine, Erasmus University Medical Centre, Department of Obstetrics and Gynaecology, P.O. Box 2040, 3000 Rotterdam, CA The Netherlands; Department of Public Health, Erasmus University Rotterdam, P.O. Box 2040, 3000 Rotterdam, CA The Netherlands; Department of Social Sciences, Erasmus University College, Erasmus University Rotterdam, PO Box 1738, 3000 Rotterdam, DR The Netherlands

**Keywords:** Pregnancy, Antenatal healthcare, Care pathway, Collaboration, Risk assessment

## Abstract

**Background:**

Promotion of healthy pregnancies has gained high priority in the Netherlands because of relatively unfavorable perinatal outcomes. In response, a nationwide study, ‘Healthy Pregnancy 4 All’ (HP4ALL), has been initiated. Part of this study involves systematic and broadened antenatal risk assessment (the Risk Assessment substudy). Risk selection in current clinical practice is mainly based on medical risk factors. Despite the increasing evidence for the influence of nonmedical risk factors (social status, lifestyle or ethnicity) on perinatal outcomes, these risk factors remain highly unexposed. Systematic risk selection, combined with customized care pathways to reduce or treat detected risks, and regular and structured consultation between community midwives, gynecologists and other care providers such as social workers, is part of this study.

**Methods/Design:**

Neighborhoods in 14 municipalities with adverse perinatal outcomes above national and municipal averages are selected for participation. The study concerns a cluster randomized controlled trial. Municipalities are randomly allocated to intervention (n = 3,500 pregnant women) and control groups (n = 3,500 pregnant women). The intervention consists of systematic risk selection with the Rotterdam Reproductive Risk Reduction (R4U) score card in pregnant women at the booking visit, and referral to corresponding care pathways. A risk score, based on weighed risk factors derived from the R4U, above a predefined threshold determines structured multidisciplinary consultation. Primary outcomes of this trial are dysmaturity (birth weight < p10), prematurity (birth <37 weeks), and efficacy of implementation.

**Discussion:**

The ‘HP4ALL’ study introduces a systematic approach in antenatal health care that may improve perinatal outcomes and, thereby, affect future health status of a new generation in the Netherlands.

**Trial registration:**

Dutch Trial Registry ( NTR-3367) on 20 March 2012.

**Electronic supplementary material:**

The online version of this article (doi:10.1186/1745-6215-16-8) contains supplementary material, which is available to authorized users.

## Background

Perinatal mortality rates in the Netherlands are higher than in other European countries and are showing a slower rate of decline [1]. The latest confirmed statistics describe a perinatal mortality rate (deaths from 22 weeks of gestation up to 7 days postpartum) of 8.5 per 1,000 births [2]. In addition, higher risks for adverse outcomes were found for women living in socioeconomically deprived areas, particularly in the four largest cities in the Netherlands [3]. The adverse perinatal health outcome in the Netherlands has triggered debates and initiatives to study and intervene in possible causes for these adverse outcomes.

More than 85 percent of all causes of perinatal mortality in the Netherlands are associated with four adverse perinatal outcomes, namely congenital disorders, small for gestational age (birth weight <10th percentile for gestational age), premature delivery (birth <37 weeks), and/or a suboptimal start at birth (Apgar score <7 after 5 minutes); these are defined as the ‘Big 4’ outcomes [4]. Poor outcomes were especially observed in deprived districts and are often associated with socioeconomic and ethnicity related risk factors such as low education, low income and poor integration into society [5, 6].

### The Dutch system

The Dutch antenatal healthcare system is divided into three levels of care in which a distinction is made between low-risk and high-risk pregnancies. The primary level of care, antenatal health care is provided by independently practicing midwives who deliver care to pregnant women with an uncomplicated pregnancy, childbirth and the postpartum period. These women are estimated to be ‘low risk’. If complications (threaten to) occur, midwives refer the women to a gynecologist at the secondary level of care [7]. The tertiary level of care takes place in centers for perinatology with a neonatal intensive care unit and an obstetric ‘high-care’ department. The latter is reserved for severely ill women or threatened pregnancies [8].

### Current practice of risk selection

Current risk selection during the antenatal period focuses on medical risk factors [9, 10]. A list of obstetric indications, for which referral from primary to secondary care is prescribed, has been composed by professionals involved in the field. On this list, the most frequently occurring medical conditions and prior obstetric complications are allocated to the different levels of care. The composition of this list was based on best evidence or best practices. Since the last update in 2003, there has been an increase in evidence of the influence of nonmedical risk factors (social status, lifestyle or ethnicity) on adverse perinatal outcomes [11]. These risk factors are usually not considered in current risk screening practices. Moreover, it was shown that an accumulation of these risk factors can even further harm the chances of a good pregnancy outcome. This phenomenon implies that the presence of a number of smaller risk factors, rather than a single greater one, increases the risk on adverse perinatal outcomes [4, 11, 12].

### Initiatives to improve perinatal health

To improve perinatal health, especially in socially deprived districts, the Rotterdam municipal council and health scientists of the Erasmus University Medical Centre initiated a city-wide perinatal health program ‘Ready for Baby’ in 2009 [5]. The ‘Ready for Baby’ program provided the framework for this national study. It was within this program that scorecard-based risk screening and corresponding care pathways were developed and piloted. Within the context of this program, the Rotterdam area served as a testing ground for a number of experiments, including the development and piloting of the R4U (Rotterdam Reproductive Risk Reduction) scorecard for antenatal risk screening [13]. These former experiences were used to further improve and implement these tools in other municipalities with high perinatal mortality and morbidity in the Healthy Pregnancy 4 All study. The Healthy Pregnancy 4 All study was launched in 2011 by the Erasmus University Medical Centre in 14 municipalities in the Netherlands. It focuses on preconception care and broadened risk selection during pregnancy [14].

In the risk assessment substudy, we will implement and investigate the Rotterdam Reproductive Risk Reduction (R4U) scorecard, corresponding care pathways and multidisciplinary consultation. This score card focuses on both medical and nonmedical risk factors, including psychological, social, lifestyle, obstetric and nonobstetric care-related risks. During the first antenatal visit, the R4U scorecard will be assessed by a midwife or gynecologist. The aim of this study is to investigate the effectiveness of this new approach in antenatal health care on perinatal outcomes.

## Methods/Design

### Outline

The study design concerns a cluster randomized controlled trial at the municipality level. Selected municipalities are randomly allocated to intervention and control groups. The intervention comprises systematic risk selection with the R4U scorecard in pregnant women at the booking visit and referral to the corresponding care pathways. A risk score, based on weighed risk factors derived from the R4U, above a predefined threshold determines consultation among community midwives, gynecologists, and other care providers, such as social workers in a multidisciplinary setting.

The study aims 1) to investigate the effectiveness of systematic approach in antenatal health care on adverse pregnancy outcomes (primarily lowering prematurity and small for gestational age outcomes) and 2) the efficacy of implementation (measured by the number of R4Us filled out by the healthcare professional, the performance of multidisciplinary deliberations and patient and healthcare professional satisfaction).

### Participants/eligibility criteria

The selection of neighborhoods is based on the presence of an elevated incidence of adverse perinatal outcomes (above both the national and municipal average). Municipalities are extracted after a selection process in which zip codes with high adverse perinatal outcomes are identified in a thorough analysis. For this analysis, data from all singleton pregnancies in the Netherlands over the period of 2000 to 2008 were obtained with permission from the Dutch Perinatal Registry (PRN) [15]. The detailed selection process of the municipalities and geographical areas are described elsewhere [14].

All midwives and gynecologists providing care to women living in these zip codes will be invited to participate in the Healthy Pregnancy 4 All study. All pregnant women living in these selected areas are eligible for this trial. All municipalities deal with an above-average perinatal mortality rate and many disadvantaged neighborhoods. Exclusion criteria include an acute obstetric situation during the booking visit (for example, ectopic pregnancy) and women in labor during this initial visit.

### Study design

To prevent contamination, we have randomized on the level of municipalities instead of (1) on the level of midwife practices or (2) on the level of hospitals or obstetric collaborations.

Randomization at the level of the individual patient is not possible because of contamination to a healthcare professional: a care provider will not be able to distinguish between intervention and control participants within their practice.

Many midwife practices are cooperating with hospitals in so-called obstetric collaborations (OC’s). Randomization on the level of OC’s was also not an option: hospitals are always involved in one particular OC, but midwife practices can participate in more than one OC. This is especially the case in municipalities with more than one hospital.

### Intervention and control

Use of the R4U scorecard, corresponding care pathways, and multidisciplinary consultation will be compared with conventional antenatal health care. In municipalities allocated to the intervention group, midwives and gynecologists will use the R4U scorecard during every first antenatal visit (provided that informed consent is given). The R4U scorecard consists of six domains (social status, ethnicity, care, lifestyle, medical history and obstetric history), subdivided into 70 items. The R4U scorecard is proposed to facilitate improved coordination of antenatal care through systematic and uniform risk screening for medical and nonmedical risk factors. A risk score, based on risk factors derived from the R4U, above the predefined cut-off point implies follow-up action. This follow-up action includes multidisciplinary consultation between obstetric caregivers and nonobstetric caregivers (for example, social workers) and prioritization of risk factors. For each cluster, this cut-off point will be calculated after a pilot of 50 R4U scorecards have been completed in a municipality. We strive to assess 20% of all pregnant women in this multidisciplinary setting. Healthcare professionals determine when and how to organize these meetings, with a minimum frequency of once a month. A standardized format is available to discuss a patient in such meetings. The purpose of these meetings is to assess a customized antenatal policy for each individual pregnant woman.

We developed 28 templates of care pathways for all risk factors incorporated in the R4U score card. Care pathways consist of steps a caregiver could follow to meet specific needs for pregnant women. Together with local healthcare professionals from both the perinatal care, community health services (called the GGD), and other services, these templates will be adapted to local circumstances. Local facilities, agreements, and guidelines will be incorporated. Depending on local availability, care pathways will developed for all items as presented in the R4U scorecard. These created care pathways will support the individual healthcare professional to encounter complex (non-) medical risk factors. They can also facilitate the collaboration among different healthcare professionals and professionals within the community health services.

Pregnant women in the control group will receive conventional antenatal health care. After the inclusion of 700 participants or after two-thirds of our study period (2 years), we will end the control group in a particular municipality and will start with the implementation of the R4U scorecard and corresponding care pathways. As mentioned in our introduction, several studies revealed a disadvantaged position of the Netherlands regarding perinatal mortality. For the first time, the unique organization of antenatal care in the Netherlands was openly questioned. In response, health care professionals and policy makers were urged to undertake interventions. The fact that we made municipalities aware of their perinatal health statistics and allocated them to be ‘a control’ was contradictory to ambitions to intervene, as the control status was felt to be the negligent attitude of local health authorities.

We are lenient to this aspect and will offer them the opportunity to start with the intervention after they have reached a certain amount of included subjects or at two-thirds of the way through the study. Therefore, we aim to implement and facilitate this new approach in all 14 municipalities at the end of the study period. This implies that the study design remains a parallel trial in which only pregnancy outcomes from regular antenatal health care in control municipalities will be analyzed. Pregnancy outcomes of women enrolled after the ‘cross-over’ in control municipalities (that is, women in control municipalities that were exposed the intervention) will not be analyzed for this purpose.

### Procedures, recruitment, randomization, and collection of baseline data

Randomization took place in January 2011 by an independent statistician who was not involved in executing the study. In order to decide whether matching was necessary, in each cluster, the adverse perinatal outcomes (Big 4 and perinatal mortality) were stratified according to socioeconomic status and ethnicity. Since clusters were not considerably different in terms of these characteristics, we decided that matching of clusters for socio-economic status and ethnicity was unnecessary.

As stated before, midwife practices participate in OC’s. Due to merging plans between OC’s in the north of the Netherlands, four small municipalities and one large municipality were combined and form one cluster (named Groningen). Thus, ten municipalities were randomly assigned to the intervention (n = 5) or the control group (n = 5).

In the randomization procedure, municipalities were numbered according to the expected number at risk. Hereby, we ensured that both arms of the experiment were comparable in size. Five random numbers were drawn from the uniform distribution between zero and one. If the number was below 0.5, the first of the pair would be assigned to the intervention, otherwise to the control group. The clusters Amsterdam, Tilburg, Groningen, Enschede and Nijmegen are allocated as intervention municipalities. The clusters for The Hague, Schiedam, Heerlen, Almere and Utrecht are control municipalities. The randomization scheme is available as an additional file (see Additional file 1).

The logistics are carried out in close collaboration with participating local program coordinators, midwives, gynecologists, and, if available, research midwives in the 14 participating municipalities. Eligible women will receive participant information, and they will be asked for written consent to collect data on their pregnancy outcomes by the participating healthcare professionals. These healthcare professionals will register all participating women anonymously by a study number in a web-based database. We will ask to register the 4-digit zip code, maternal age, gestational age at booking visit, and certain items of the obstetric history. All women participating in the risk assessment study within the HP4ALL study will receive either one or two questionnaires. The first questionnaire contains questions regarding baseline characteristics, such as marital status, household composition and income, education level, ethnicity, lifestyle (smoking, alcohol, and drug use), use of folic acid, medical history, and use of medication. This questionnaire will be completed around the first antenatal visit (depending on local logistics). The second questionnaire focuses on satisfaction. In this questionnaire, we will investigate patient satisfaction regarding their first antenatal visit. We compare satisfaction in participants in the intervention group with participants receiving conventional antenatal health care. This questionnaire will be completed by a selection of study participants. We will ask all participating practices/hospitals to distribute this questionnaire in a predefined time period of 3 months to all study participants having their third antenatal visit. This visit is before the so-called 20-week ultrasound. With this timing, we try to avoid bias in answering the questionnaire due to outcomes resulting from this ultrasound.

To provide baseline characteristics from participating healthcare professionals, an interview will be performed with one healthcare professional from each participating midwifery practice and hospital. In this structured interview, we will collect data on the number of patients and employees, the current use of risk-selection instruments, collaboration with hospitals and (other) midwifery practices in OC’s, and work processes in their antenatal healthcare (for example, counseling for prenatal screening, or ultrasound facilities). This will be repeated at the end of the study. We will use the Relational Coordination survey to measure the coordination performance of healthcare professionals. Gittell’s theory of Relational Coordination has been developed for measuring coordination performance among different professions. The healthcare professionals will be surveyed about their communication and relationships with other healthcare professionals during the antenatal phase. This will allow us to observe the effect of our intervention on the coordination performance of healthcare professionals [16]. Table 1 provides an overview of the planned assessments within the risk assessment substudy including variables, methods and outcomes.

**Table 1:** **Planned assessments of the risk selection experiment: variables, methods and outcomes**

Patients		
Variables	Methods	Outcomes
1. INTERVENTION GROUP		
Nonmedical risk factors: 39 items from the risk scorecard, categorized into the domains of social, ethnicity, care, and lifestyle.	R4U scorecard + registration form ‘Obstetric history’	Primary outcomes:
		- Preterm birth
		- Small for gestational age
Medical risk factors: 30 items from the risk scorecard, categorized into the domains of general history and obstetric history	Questionnaire ‘Baseline characteristics’	Secondary outcomes - Undetected small for gestational age and unexpected preterm births (babies born in the first level of care)
Baseline characteristics: Age, zip code, ethnicity, onset of care, household composition, family income, employment, education level, smoking, alcohol, drugs, folic acid use, medication use, pre-existing chronic diseases, and sexually transmitted diseases.	Case Record Form ‘pregnancy and delivery data’	- Prevalence of risk factors
		- Risk accumulation
		- Involved healthcare professionals during pregnancy
		- Detection and prevention of impaired growth and preterm birth during pregnancy
		- Perinatal mortality
		- Congenital anomalies
		- Delivery modus
		- Place of delivery
2. CONTROL GROUP		
Baseline characteristics: Age, zip code, ethnicity, onset of care, household composition, family income, employment, education level, smoking, alcohol, drugs, folic acid use, medication use, pre-existing chronic diseases, and sexually transmitted diseases.	Registration form ‘Obstetric history’	- Asphyxia
	Questionnaire ‘Baseline characteristics’ Case Record Form ‘pregnancy and delivery data’	- Neonatal admission
		- Maternal morbidity (such as pre-existing chronic disease, pregnancy complications, positive booking bloods), and maternal mortality.
Patient satisfaction in both groups	Questionnaire ‘Patient experiences during the first antenatal visit’	- Which topics were discussed (10 examples)?
		- What was your experience?
		- Do you think this was important to ask?
**Care providers**		
**Variables**	**Methods**	**Outcomes**
General characteristics participating midwives practices and hospitals in both groups	Interview-based questionnaire	- Current status number of patients and employees
		- Use of risk selection instruments
		- Collaboration with hospitals and (other) midwifery practices
		- Work processes (for example, counseling for prenatal screening, or ultrasound facilities)
Care provider satisfaction in both groups	Questionnaire	- Feasibility
		- Efficacy of implementation
		- Collaboration
		- Continuation of intervention

### Outcome

The primary outcomes are small for gestational age, preterm birth, and efficacy of implementation. Small for gestational age is defined as birth weight below the 10th percentile for gestational age. Preterm birth is defined as birth before 37 weeks of gestation.

Efficacy of implementation will be measured by the number of R4Us completed by the healthcare professional against the number of booking visits, the development and use of care pathways, actual performed multidisciplinary consultations, and patient and healthcare professional satisfaction. Primary outcomes, except for patient satisfaction, will be recorded by healthcare professionals or the project team after delivery on case record forms in an electronic database.

Secondary outcomes are perinatal mortality (from the 22th week of gestation until 7 days postpartum), undetected small for gestational age and unexpected preterm births (babies born in the first level of care), and prevalence and accumulation of medical and nonmedical risk factors.

Other outcome parameters are: detection and prevention of impaired growth and preterm birth during pregnancy, delivery modus, place of delivery, involved healthcare professionals, congenital anomalies, neonatal admission, asphyxia, maternal morbidity (such as pre-existing chronic disease, pregnancy complications, and positive booking bloods) and maternal mortality.

### Follow-up

The follow-up period consists of 6 weeks. Details of pregnancy, delivery, and maternal follow-up will be recorded after 6 weeks in a case record form. If necessary, medical records of newborns will be requested (if consent is provided).

### Statistics

#### Sample size

The sample size is determined by focusing on the primary objective of the trial: the effectiveness of systematic antenatal screening on adverse small for gestational age (<10th percentile) and preterm birth (gestational age below 37 weeks). In this cluster-randomized trial, sample size depends on (1) the average risk of small for gestational age and preterm birth without the intervention (π_0_); (2) the expected effect of the intervention (π_1_); (3) the inflation factor reflecting the partial similarity/dependency of women’s outcomes or responses within the same cluster; (4) the α; and (5) power (1-β) of the test.

Based on data from the Dutch perinatal registry [15], the average prevalence in our selected zip code areas in the years 2000 to 2008 was 16.7%. In the intervention group, we expect a prevalence of 13% at the end of the study period. The average prevalence of both primary outcomes in the Netherlands was 12% for the years 2000 to 2008 [15], and we expect a decline towards this prevalence during our study period. The inflation factor was estimated to be 2.06 [17]. This factor reflects the design effect of a cluster trial, and takes into account the degree of similarity among responses within a cluster. With five municipalities in each group (a total of ten clusters) and an alpha of 0.05 (two-sided test), 7,000 participants (3,500 per arm) should provide power in excess of 80%. This means 700 women are needed per cluster. It is assumed that a difference of 150 to 200 participants between the larger and smaller clusters has no implications for the outcome [17].

#### Data analysis

Data will be analyzed according to the intention-to-treat principle. The effectiveness of the R4U scorecard, corresponding care pathways and multidisciplinary consultation versus conventional antenatal healthcare (measured as the difference in small for gestational age and preterm birth between both groups) will be assessed by using multilevel logistic regression with random effects. Results will be presented as effect estimates with a measure of precision (95% confidence interval). Data will be analyzed anonymously on two levels: the maternal and the municipal level. To guarantee anonymity, we will not analyze the data on community practice or at the hospital level. Study participants, municipalities, and community practices or hospitals will not be traceable. Other analyses related to our intervention are based on differences in other birth outcomes (for example, Apgar score), the number of referrals between community midwives and gynecologists, adequacy in risk assessment (for example, detection of growth restriction in the antenatal phase), and the contribution of nonmedical risk factors to adverse birth outcomes between the intervention and control group. These outcomes will be assessed with univariate and multivariate regression analysis with 95% confidence intervals.

### Ethical considerations

This study has been approved by the Ethics Committee of the Erasmus Medical Center Rotterdam (Ref. No. MEC-2012-322) and by the management of participating hospitals that requested an extra review (see Additional file 2).

All participants will receive written and oral information about the study, after which informed consent will be obtained. Participation is voluntary and no extra incentives will be provided.

## Discussion

The main objective of this trial is to investigate the effectiveness of a new systematic approach in antenatal healthcare on adverse pregnancy outcomes and efficacy of implementation. We will implement and investigate the Rotterdam Reproductive Risk Reduction (R4U) scorecard and corresponding care pathways in 14 municipalities.

The study meets the current evidence to intervene early in pregnancy upon (modifiable) risk factors associated with adverse perinatal health outcomes. We aim to target a population that potentially will benefit the most with the use of selected geographical areas. With the use of care pathways, optimal linkage is sought between curative and preventive care, such as public health, government, and social welfare organizations [14].

To our knowledge, this is the first approach in antenatal healthcare whereby systematic risk selection for both medical and nonmedical risk factors, variable thresholds, tailor-made care pathways, and structured consultation between midwives, gynecologists and other care providers are combined. This study will introduce a systematic approach in antenatal health care, which may improve perinatal outcomes and, thereby, future health status of a new generation in the Netherlands.

### Trial status

Recruitment started in August 2012, and the first included study participant delivered in March 2013. The trial is currently recruiting study participants.

## Electronic supplementary material

Additional file 1: **Randomization procedure.** (DOCX 15 KB)

Additional file 2: **Ethical bodies that approved the study.** (DOC 30 KB)

## Abbreviations

HP4ALL: Healthy Pregnancy 4 ALL
OC: obstetric collaboration
PRN: Perinatal Registry Netherlands
R4U: Rotterdam Reproductive Risk Reduction.
