# Supplementary material for: Effectiveness of score card-based antenatal risk selection, care pathways, and multidisciplinary consultation in the Healthy Pregnancy 4 All study (HP4ALL): study protocol for a cluster randomized controlled trial
Source: Trials. 2015 Jan 6;16:8. doi: 10.1186/1745-6215-16-8 (PMC4326478; doi:10.1186/1745-6215-16-8)
Supplement: Supplementary file 1 — Additional file 1: Randomization procedure. (DOCX 15 KB) [file 13063_2014_2394_MOESM1_ESM.docx]

Additional file 1: Randomisation procedure

25 januari, 2012CL

Randomizing municipalities

This is a description of the randomization procedure for assigning 10 municipalities to intervention

or control group. It was thought cautious to match municipalities on size, as to prohibit all small

municipalities to be in one arm. Therefor the municipalities were numbered according to the

expected number at risk.

The principal researcher then was asked to choose an integer number from 50 provided to her,

in order to _x the seed for the random number generator. The whole procedure was performed in

R and the result listed at the end of this message.

Five random numbers were drawn from the uniform distribution between zero and one. If the

number was below 0.5 the _rst of the pair would be assigned to the intervention, otherwise to the

control group.

The result is that numbers 1, 4, 6, 7 and 9 became the intervention group, the others the control

C.W.N. Looman

statistician

R version 2.7.1 (2008-06-23)

Copyright (C) 2008 The R Foundation for Statistical Computing

ISBN 3-900051-07-0

R is free software and comes with ABSOLUTELY NO WARRANTY.

You are welcome to redistribute it under certain conditions.

Type 'license()' or 'licence()' for distribution details.

R is a collaborative project with many contributors.

Type 'contributors()' for more information and

'citation()' on how to cite R or R packages in publications.

Type 'demo()' for some demos, 'help()' for on-line help, or

'help.start()' for an HTML browser interface to help.

Type 'q()' to quit R.

> matrix(round(runif(50,1,1000)),nrow=5)

[,1] [,2] [,3] [,4] [,5] [,6] [,7] [,8] [,9] [,10]

[1,] 356 758 823 18 902 394 563 881 272 468

[2,] 805 542 890 332 687 192 907 215 587 510

[3,] 234 469 587 502 557 741 894 472 859 123

[4,] 729 898 232 922 206 949 63 435 994 957

[5,] 753 330 504 33 984 592 161 241 324 915

> rm(list=ls(all=TRUE)) # clear the memory

> options(digits=4) # options for number of significant digits > options(width=158)

>

> set.seed(949)

>

> nr <- 1:10

> paar <- rep(1:5,each=2)

> cbind(nr,paar)

1

nr paar

[1,] 1 1

[2,] 2 1

[3,] 3 2

[4,] 4 2

[5,] 5 3

[6,] 6 3

[7,] 7 4

[8,] 8 4

[9,] 9 5

[10,] 10 5

>

> ran01 <- runif(5)

> ran01

[1] 0.46505 0.77163 0.95071 0.45522 0.01422

> ran01>0.5

[1] FALSE TRUE TRUE FALSE FALSE

>

> 1+ifelse(ran01>0.5,1,0)+(0:4)*2

[1] 1 4 6 7 9

>

> interv <- rep(FALSE,10)

> interv[1+ifelse(ran01>0.5,1,0)+(0:4)*2] <- TRUE

> cbind(nr,paar,interv)

nr paar interv

[1,] 1 1 1

[2,] 2 1 0

[3,] 3 2 0

[4,] 4 2 1

[5,] 5 3 0

[6,] 6 3 1

[7,] 7 4 1

[8,] 8 4 0

[9,] 9 5 1

[10,] 10 5 0

2
