# Supplementary material for: Effectiveness of score card-based antenatal risk selection, care pathways, and multidisciplinary consultation in the Healthy Pregnancy 4 All study (HP4ALL): study protocol for a cluster randomized controlled trial
Source: Trials. 2015 Jan 6;16:8. doi: 10.1186/1745-6215-16-8 (PMC4326478; doi:10.1186/1745-6215-16-8)
Supplement: Supplementary file 2 — Additional file 2: Ethical bodies that approved the study. (DOC 30 KB) [file 13063_2014_2394_MOESM2_ESM.doc]

Names of ethical bodies that approved your study

General approval for the study was obtained from the Medical Ethical Board of the Erasmus Medical Center. Ethical boards of hospitals have the opportunity to review the study protocol again. However, this is not mandatory according to the Dutch law. The following participating centers took an extra review:

| **Municipality** | **Ethical board** | **Conclusion** |
| --- | --- | --- |
| Enschede | Medisch spectrum twente | Permission |
| The Hague | Haga ziekenhuis | Permission |
| The Hague | Medisch Centrum Haaglanden | Permission |
| Utrecht | Universitair medisch centrum | Permission |
| Tilburg | Elisabeth ziekehuis | Permission |
| Heerlen | Atrium ziekenhuis | Permission |
| Nijmegen | Canisius Wilhelmina ziekenhuis | Permission |
